# Extraction of Placenta in Abortion

**Published:** 1875-04

**Authors:** 


Extraction of the Placenta in Abortion.—In the *Obstetrical Society, of Boston*, November 14, 1874, Dr. Fifield said, with reference to removing placental masses, he never could do anything with the placental forceps, but had always succeeded by the method taught him by Dr. Miller, namely, to put the patient on her back with her feet in chairs, then to place one hand upon the hypogastrium, and with the index finger of the other hand to hook down the uterus. Having on one occasion passed the forceps through a long, narrow os, he was afraid to withdraw them in position, convinced that in so doing he should split the cervix. In this case there had been no show from May till August; then nothing till the last week in October, when he saw the patient after a hæmorrhage of four or five days. He extracted a placental mass having a central cavity, which held a fœtus of six weeks' growth. The question there was whether an intercurrent hæmorrhage marked the point of death of the fœtus. In this case apparently it did not do so.—*Boston Med. and Surg. Jour., Jan.* 28, 1875, p. 103.

